# Association Between Urinary Symptoms and Urinary Tract Infection in Patients With Multiple Sclerosis

**DOI:** 10.5539/gjhs.v8n4p253

**Published:** 2015-09-28

**Authors:** Alireza Nikseresht, Haideh Salehi, Amin Abolhasani Foroughi, Masoume Nazeri

**Affiliations:** 1 Clinical Neurology Research Center, Department of Neurology, Shiraz University of Medical Sciences, Shiraz, Iran; 2 Medical Imaging Research Center, Shiraz University of Medical Sciences, Shiraz, Iran

**Keywords:** MS, multiple sclerosis, urinary symptom, UTI, urinary tract infection

## Abstract

**Background & Objective:**

Urinary dysfunctions occur in the majority of MS patients and these patients are at higher risks of developing UTI due to multiple reasons. We determined to study the association between different urinary symptoms and UTI in MS patients.

**Material & Method:**

Eighty seven MS patients that referred to our medical care center with an acute attack of the disease, from November 2012 to April 2014, were included in the study. Patients were classified into two groups based on their urine culture results UTI positive and non-UTI patients. The prevalence of different types of urinary symptoms was then compared among the two groups.

**Result:**

The mean age of our patients was 36.8 years old. From the total 87 patients, 83 (95.4%) were female. Overall 56.3% of patients displayed urinary symptoms. The most prevalent urinary problems were urinary incontinence and frequency (25.3% and 24.1%, respectively). A positive urinary culture was seen in 71.3% of the patients. The prevalence of urinary problems was significantly higher in UTI patients in comparison to non-UTI patients (64.5% and 40% in UTI and non-UTI patients, respectively; p=0.036). Separately none of the different urinary symptoms displayed a significant difference between UTI and non-UTI patients (p>0.05).

**Conclusion:**

Not a single symptom can be diagnostic of UTI, but MS patient with urinary tract infections do present more urinary symptoms and this can be an indication for further urine analysis and screening measures for MS patients who display more urinary symptoms.

## 1. Introduction

Multiple sclerosis (MS) is a demyelinating disease of the central nervous system (CNS) that presents with a progressive and varying clinical course (Compston & Coles, 2002; Noseworthy, Lucchinetti, Rodriguez, & Weinshenker, 2000). The disease affects 1 in every 1000 people in the US (Nicholas, Young, & Friede, 2010), and is three times more prevalent in women than men. It is the most prevalent neurological disease affecting people between the ages of 20-45 years old (Fingerman & Finkelstein, 2000).

The disease is associated with a wide range of symptoms. The symptoms are the result of focal demyelination lesions at different levels of the central nervous system. Based on the location and the different characteristics of the plaques in the CNS and the concomitant edema, MS patients report different neurological and urological symptoms, over the course of the disease (Ciancio, Mutchnik, Rivera, & Boone, 2001; Litwiller, Frohman, & Zimmern, 1999; Philp, Read, & Higson, 1981).

Most of MS patients develop lower urinary symptoms, a condition referred to as a *Neurogenic Bladder* (de Almeida, Carneiro, Fiorelli, Orsini, & Alvarenga, 2013; de Seze, Ruffion, Denys, Joseph, & Perrouin-Verbe, 2007; Fowler et al., 2009). Urinary related symptoms occur in about 50-80% of MS patients and can result in significant morbidity and dysfunctions (Aksungar, Topkaya, Yildiz, Sahin, & Turk, 2008). The etiology of this condition is attributed to a disco ordination among the brainstem centers and the sacral parts of the spinal cord (Fowler et al., 2009).

Previous reports have described several disturbances during urodynamic investigation such as detrusor hyper-reflexia or hypo-reflexia and detrusor sphincter dysynergia, with a poor correlation with the clinical signs (Barbalias, Liatsikos, Passakos, Barbalias, & Sakelaropoulos, 2001; Onal, Siva, Buldu, Demirkesen, & Cetinel, 2009).

Because of some of the urinary problems that MS patients face, especially urinary incontinence, urinary stasis and the use of catheters, they are at higher risks of developing urinary tract infections (UTI), which can lead to systemic infections and sepsis if left untreated (Mahadeva, Tanasescu, & Gran, 2014). These infections can even affect the upper urinary tract and have insidious consequences for these patients including permanent kidney damage.(Metz, McGuinness, & Harris, 1998)

High dose administration of steroids with a concomitant infection may exacerbate the infection (Mahadeva et al., 2014). One study (Kupelian et al., 2013) documented that 4 hours after urine sample collection, cell loss with and without the use of preservatives was 40% and 60%, respectively, which can indicate that some lower grade infections are undetected and in result untreated due to the occurring cell loss. The presenting symptoms of UTI may be confused with the pre-existing urinary problems in MS patients, thus making the diagnosis of urinary infections more difficult in patients with MS, furthermore the urgency of early diagnosis of UTI emphasizes the importance of detecting these patients before the onset of severe symptoms. So we determined to study the association between different urinary symptoms and UTI in MS patients.

## 2. Material and Method

### 2.1 Study Design and Patient Selection

This is a cross-sectional study, conducted in Chamran hospital in Shiraz, Iran. The study protocol was approved by both the Ethics Committee and Institutional Review Board of Shiraz University of Medical Sciences. All the patients gave their written consent form to enter the study.

All MS patients that referred to the hospital with an attack of the diseases and required a pulse therapy of methyl prednisolone, from November 2012 to April 2014 were included in the study.

Our exclusion criteria was: having other diseases that could affect the test results like Parkinson’s disease, spinal cord injuries, stroke, diabetes, having a history central nervous system dysfunctions other than MS and using any drugs that could influence the infection or the results of the urine culture.

### 2.2 Data Collection

Patients information regarding age, sex, MS type, urinary symptoms including: urinary retention, incontinence, urgency, frequency, dribbling, dysuria and hesitancy, were registered. The history of all the patients was obtained and later a neurological and urological examination was performed for all the patients. Based on patients’ history of disease and physical findings they were classified into three groups: relapsing-remitting, secondary progressive and patients that had a newly diagnosed MS on referral. Before administrating the required therapy for their acute attack, a urine sample was collected from all the patients and was tested for bacterial growth.

The reason for obtaining the culture as a routine procedure in MS patients with an acute attacks prior to the administration of immuno-suppressive drugs is that in case of an infection, by administering these drugs, patients would face a more severe and even a systemic spread of infection.

Patients were classified into two groups based on their urine culture results: those that had positive urine cultures (UTI positive) and those that had a negative urine culture (non-UTI). The prevalence of different types of urinary symptoms was then compared among the two groups.

### 2.3 Definition of Variables

The relapsing-remitting type of MS was defined as having clear relapses with full recovery or with sequelae and residual deficit on recovery. The periods between disease relapses were characterized by a lack of disease progression. The secondary progressive type was defined as an initial relapsing-remitting disease course followed by progression with or without occasional relapses, minor remissions and plateaus. The newly diagnosed patients were those patients that got their initial diagnosis of MS at the time in which they referred to our medical care center.

Regarding the urinary symptoms, urgency was considered as a prominent desire to urinate. Urinary frequency as urinating more than eight times in a day. Dysuria as pain during urination. Urinary hesitancy as a delay in initial passage of urine. Incontinence as the loss of bladder control. Retention as the inability to pass urine and post void dribbling was defined as the leakage of a small volume of urine immediately or shortly after completing urinating.

After acquiring a culture from the patients’ urine samples, a positive urine culture was defined as the presence of ≥10^4^ colony forming units or the presents of ≥10^5^ mixed growth with the predominance of one organism or the existence of ≥10^3^ of *E. Coli* or *Staphylococcus Saprophyticus*. The presence of ≥10 white blood cells in the urine analysis was also considered as a sign of infection.

### 2.4 Statistical Analysis

Data was analyzed using the Statistical Package for Social Sciences software, SPSS for windows, version 20 (SPSS Inc., Chicago, IL, USA). Patients’ data are presented as frequency and percent. The Chi-square test was used to compare the data between the groups. A two-tailed p-value of less than 0.05 was considered as statistically significant.

## 3. Result

Eighty seven patients entered the study. The mean age of our patients was 36.8 years old. From the total 87 patients, 83 (95.4%) were female. MS types were classified into three groups as mentioned before. Analysis showed that the most prevalent type of MS among our patients was the relapsing remitting form of disease (46%) and after that the secondary progressive and the newly diagnosed MS (34% and 19%, respectively).

**Figure 1:**
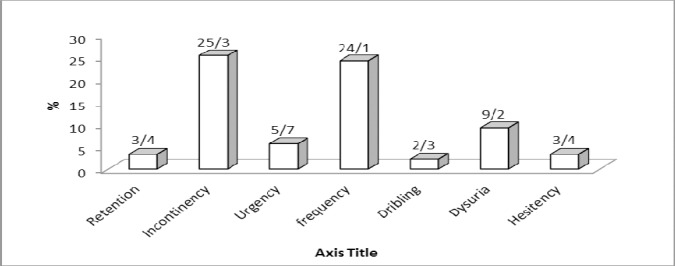
The diagram displays the frequency of different urinary symptoms in the patients

Regarding urinary problems overall 56.3% of patients displayed urinary symptoms. The most prevalent urinary problems were urinary incontinence and frequency (25.3% and 24.1%, respectively). Overall 3.4% patients displayed signs of retention, 25.3% showed incontinence. Urinary urgency, frequency, dribbling, dysuria and hesitancy were prevalent in 5.7%, 24.1%, 2.3%, 9.2% and 3.4% of the patients, respectively (Figure 1).

Urinary tract infection or a positive urinary culture was seen in 71.3% of the patients (Table 1).

**Table 1:** Patients’ baseline and disease related characteristics.

characteristic	Frequency – no. (%)
Sex	
Male	4 (4.6)
Female	83 (95.4)
overall	87 (100)
Age – years	36.8 ± 9.9
Urinary problem*	
Positive	49 (56.3)
negative	38 (43.7)
MS type	
Relapsing remitting	40 (46)
Chronic progressive	30 (34.5)
Newly diagnosed	17 (19.5)
Urinary problem type	
Incontinence	22 (25.3)
frequency	21 (24.1)
Retention	3 (3.4)
Urgency	5 (5.7)
Dribbling	2 (2.3)
Dysuria	8 (9.2)
Hesitancy	3 (3.4)
UTI ¶	
Positive	62 (71.3)
negative	25 (28.7)

UTI: Urinary tract infection;

* A positive urinary problems was considered as having any of the urinary symptoms.

Presents of ≥10^4^ colony forming units or ≥10^5^ mixed growth with the predominance of one organism or ≥10^3^ of *E. Coli* or *Staphylococcus Saprophyticus* or ≥10 white blood cells in the urine analysis were considered as positive UTI.

As seen in the figure urinary incontinence and urinary frequency were the most common type of urinary symptom seen among MS patients.

Urinary symptoms were compared between the UTI and non-UTI patients. In the UTI patients, 64.5% displayed urinary problems, meanwhile 40% of non-UTI patients showed urinary problems. The prevalence of urinary problems was significantly higher in UTI patients in comparison to non-UTI patients (64.5% and 40% in UTI and non-UTI patients, respectively; p = 0.036).

Urinary incontinence was the most prevalent sign in non-UTI patients (25.8% and 24% positive in UTI and non-UTI patients, respectively). Urinary frequency presented as the most prevalent symptom in UTI patients (27.4% and 16% in UTI and non-UTI patients, respectively).

Different urinary problems were compared between the two groups. Our results did not show a meaningful difference between the UTI and non-UTI patients in the prevalence of urinary incontinence and urinary frequency (p = 0.861 and p = 0.26, respectively).

Non-UTI patients did not display signs of urinary retention, urgency, dribbling and hesitancy and these symptoms were only seen in UTI patients, furthermore only 1 patients, out of the 25 non-UTI patients, showed signs of dysuria.

The prevalence of none of these types of urinary problems was statistically different between UTI and non-UTI patients (p = 0.263, p = 0.144, p = 0.364, p = 0.287 and p = 0.263 for retention, urgency, dribbling, dysuria and hesitancy, respectively) (Table 2).

**Table 2:** Comparison of urinary symptoms between UTI and non-UTI patients

Urinary symptoms*	UTI – no. (%)			Non-UTI – no. (%)			p-value
					
	Urinary sign positive	Urinary sign negative	Overall	Urinary sign positive	Urinary sign negative	Overall	
Incontinence	16 (25.8)	46 (74.2)	62 (100)	6 (24)	19 (76)	25 (100)	0.861
Frequency	17 (27.4)	45 (72.6)	62 (100)	4 (16)	21 (84)	25 (100)	0.26
Retention	3 (4.8)	59 (95.2)	62 (100)	0 (0)	25 (100)	25 (100)	0.266
Urgency	5 (6.1)	57 (91.9)	62 (100)	0 (0)	25 (100)	25 (100)	0.146
Dribbling	2 (3.2)	60 (96.8)	62 (100)	0 (0)	25 (100)	25 (100)	0.364
Dysuria	7 (11.3)	55 (88.7)	62 (100)	1 (4)	24 (96)	25 (100)	0.287
Hesitancy	3 (4.8)	59 (95.2)	62 (100)	0 (0)	25 (100)	25 (100)	0.263
Overall	40 (64.5)	22(35.5)	62 (100)	10 (40)	15 (60)	25 (100)	0.036

UTI: Urinary tract infection;

* Urgency was considered as a prominent desire to urinate. Urinary frequency as urinating more than eight times in a day. Dysuria as pain during urination. Urinary hesitancy as a delay in initial passage of urine. Incontinence as the loss of bladder control. Retention as the inability to pass urine and post void dribbling was defined as the leakage of a small volume of urine immediately or shortly after completing urinating.

Dysuria as pain during urination. Urinary hesitancy as a delay in initial passage of urine. Incontinence as the loss of bladder control. Retention as the inability to pass urine and post void dribbling was defined as the leakage of a small volume of urine immediately or shortly after completing urinating.

## 4. Discussion

In this study we evaluated the prevalence and frequency of different urinary symptoms in UTI and non-UTI MS patients. The majority of our patients were female, this finding is expected since MS mostly affects females in comparison to males (Duquette et al., 1992; Orton et al., 2006).

The most common urinary symptoms that the patients complained of were urinary incontinence and frequency. In the study by Anderson et al.(Andersen & Bradley, 1976) they found that 96% of their MS patients had urinary symptoms, which was higher than that documented in our study (96 *vs*. 56.3%). Nakipoglu et al. (Nakipoglu et al., 2009) in 2008, studied 52 MS patients and investigated the prevalence and complications of urinary symptoms. They found that the prevalence of urinary symptoms among these patients was 80.8% and the most prevalent urinary symptoms were urgency (65%) and frequency (44%), which was higher than our study.

They also documented that urinary infections were the most common urinary complication in the follow-up or history of the patient (15%). Meanwhile in our study a higher rate of urinary infections were documented (71.3%).

In the study by Rakusa et al. (Rakusa et al., 2013) a total of 249 MS patients that required corticosteroid therapy were studied. All the patients were tested for urinary colonization using a urinary dipstick. They found that 11% had significant bacteriuria. Although this study was conducted among patients that had MS with an acute relapse.

We documented urinary incontinence followed by urinary urgency to be the most prevalent urinary symptoms in MS patients. De Almeida et al. (2013) in his study of 61 MS patients in 2013, found that incontinence, urgency and urinary hesitation to be the most prevalent urinary symptoms, respectively. Without considering the prevalence of urinary hesitancy their result was completely similar to our finding.

As our main goal we compared the prevalence of urinary symptoms between patient with positive and negative cultures (UTI). Not much literature has evaluated this difference among these two groups of patients. We found a significant difference between UTI and non-UTI patients regarding their prevalence of urinary symptoms. UTI positive patient displayed a higher rate of urinary symptoms. In the study by de Almeida et al. (de Almeida et al., 2013) they found that abnormal urine analysis was higher in patients with urinary symptoms in comparison to patients without urinary symptoms, although unlike our results, this difference was not statistically significant in their study (44.4% vs. 18.8; p = 0.069).

The diagnosis of UTI in MS patients is more difficult in comparison to the normal population and this is due to multiple reasons, so utilizing every sign and symptom in order to guide us towards the infection before it causes irreversible damage is of vital importance. Based on the results in our study we found that not a single symptom can be diagnostic of UTI, but MS patient with urinary tract infections do present more urinary symptoms and this can be an indication for further urine analysis and screening measures.

This study had some limitations first of all we only included patients that referred with a recurrent attack and other MS patients that might have had urinary symptoms but did not have a recurrent attack and did not refer to the medical center were not included in the study. Patients’ MS types were not considered as an independent factor and the urinary signs were only compared in the groups based on their urine cultures which might have affected the results and also urodynamic evaluations and sonography could have been helpful in further evaluating the patients.

Multifactorial studies should be conducted in larger populations that consider different MS types and evaluate the relationship between the urinary symptoms and the urine culture.
